# Cellular immune response during BK polyomavirus infection in living donor kidney transplant recipients

**DOI:** 10.1186/s12865-026-00912-x

**Published:** 2026-09-26

**Authors:** Camilla Lorant, Britt-Marie Eriksson, Torsten Eich, Bengt von Zur-Mühlen, Gabriel Westman, Anna Wiberg

**Affiliations:** 1 Department of Medical Sciences, Section of Infection Medicine, Uppsala University, Uppsala, SE-751 85 Sweden; 2 Department of Immunology, Genetics and Pathology, Uppsala University, Uppsala, Sweden; 3 Department of Surgical Sciences, Section of Transplantation Surgery, Uppsala University, Uppsala, Sweden; 4 Department of Laboratory Medicine, Institute of Biomedicine, Sahlgrenska Academy, University of Gothenburg, Gothenburg, Sweden; 5 Department of Clinical Immunology and Transfusion Medicine, Sahlgrenska University Hospital, Gothenburg, Sweden

**Keywords:** BK polyomavirus, Kidney transplantation, Immune risk profile, CD4/CD8 ratio, Lymphocyte subsets, Immunophenotyping

## Abstract

**Background:**

BK polyomavirus (BKPyV) is a significant cause of graft impairment and loss following kidney transplantation. Early prediction of which patients are at risk of BKPyV replication and BKPyV-associated nephropathy (BKPyVAN) would be beneficial, preferably prior to transplantation. From an immunological perspective, determinants of susceptibility to BKPyV reactivation remain incompletely understood. The aim of this study was to evaluate the prognostic value of lymphocyte subset counts, CD4/8 T cell ratio, and changes in CD4 + T cell counts over time. Additionally, the concept of Immune Risk Profile (IRP), defined as CD4/CD8 ratio combined with cytomegalovirus (CMV) seropositivity, was evaluated as an immunological tool to estimate the risk of developing a clinically significant BKPyV replication.

**Methods:**

This study was part of a single-centre prospective cohort including adult kidney transplant recipients between 2016 and 2018 at Uppsala University Hospital, Sweden. All recipients were routinely screened for BKPyV DNA in plasma and urine every 3 months for up to 18 months after transplantation. Immune cell phenotyping was performed using flow cytometry on blood samples collected from both living donors and recipients at the time of transplantation, as well as from recipients during follow-up.

**Results:**

In 21 of the 68 recipients (31%), BKPyV replication was detected in blood and/or urine; 11 (16%) had DNAemia. Recipients who later developed BKPyV replication had lower CD4/CD8 ratios before transplantation compared to those who remained BKPyV replication negative. Seventeen recipients (25%) met the criteria for IRP-positivity, of whom 9 (53%) developed BKPyV replication, indicating a higher risk compared to the IRP-negative recipients (OR 3.66, 95% CI 1.08–10.79; *p* = 0.034). The total T cell counts, as well as CD4 + and CD8 + T cell counts, increased 1–2 months post-transplantation compared with pre-transplant levels in both recipients who developed BKPyV replication and those who did not.

**Conclusions:**

Recipients with a low CD4/CD8 ratio and IRP-positive status were more likely to develop subsequent BKPyV replication, supporting a role for T cell imbalance in susceptibility to viral reactivation. However, the observed differences were modest and substantial overlap between the groups limits the clinical utility of these markers for individualizing BKPyV.

**Supplementary Information:**

The online version contains supplementary material available at https://doi.org/10.1186/s12865-026-00912-x.

## Introduction

BK polyomavirus (BKPyV) is a significant cause of graft impairment and loss following kidney transplantation. Primary BKPyV infection typically occurs in childhood [1], after which the virus establishes latency in the uroepithelial cells of the urinary tract [2]. Seroprevalence rates exceed 90% in most adult populations [3, 4]. During kidney transplantation, BKPyV can be transmitted to recipients whether they are seronegative or already harbour the virus. Transplant-related immunosuppression increases the risk of both donor-derived viral transmission and reactivation of latent or slowly replicating virus in the recipient.

BKPyV replication may lead to DNAemia, which affects 10–20% of kidney transplant recipients during the first year after transplantation [5, 6], and may progress to BKPyV-associated nephropathy (BKPyVAN) in approximately 1–10% of cases [1, 7–9]. Currently, the most effective treatment strategy involves reducing immunosuppression, guided by regular monitoring of BKPyV DNA levels in blood or plasma after transplantation.

Predicting which patients will develop DNAemia and BKPyVAN before transplantation could help optimize screening and reduce unnecessary testing. This is particularly relevant for recipients of organs from living donors since previous studies have indicated lower rates of DNAemia and BKPyVAN in these patients compared to recipients of organs from deceased donors. In these two studies, the 1-year incidence of BKPyVAN in recipients from living donors was 2.9% and 1.4%, respectively, versus 4.2% and 6.1% in recipients of organs from deceased donors [10, 11]. These differences raise the possibility that uniform post-transplant screening strategies may lead to over-monitoring in lower-risk populations. However, more refined risk stratification tools are needed to identify individuals within this group who are at increased risk of BKPyV replication. The living donor setting provides a suitable framework for evaluating such approaches due to its clinical and logistical standardization.

The role of the humoral immune response and BKPyV-specific antibodies in protecting against BKPyV infection is supported by studies showing that seronegative children face a higher risk of developing BKPyVAN compared to seropositive individuals [12]. However, the presence of BKPyV-specific antibodies alone does not provide sufficient protection against BKPyV reactivation and associated disease [13]. The cellular immune response appears to play a more central role than the humoral. Low absolute counts of immune cells (total T cells < 1000/µL, CD8 + T cells < 100 /µL, CD4 + T cells < 50/µL, and NK cells < 50/µL) have been shown to predict opportunistic infections [14–16]. Furthermore, studies have demonstrated that control of BKPyV replication in kidney transplant recipients correlates with the emergence and levels of BKPyV-specific cellular immune response [9, 17, 18]. This is further supported by observations that patients who rapidly develop BKPyV-specific T cell responses have self-limiting BKPyV reactivation without requiring therapeutic interventions [19]. Additionally, a decrease in BKPyV- and CMV-specific T cells from pre-transplantation to 1–2 months post-transplantation has been associated with an increased risk of BKPyV and CMV infections, respectively [20, 21]. These findings support the potential utility of measuring BKPyV-specific T cells to predict the risk of viral reactivation and BKPyVAN [22]. However, the methodology is complex [23] and not in clinical use in Sweden. Therefore, the aim of this study was to evaluate alternative predictive markers among analyses that are, or could easily be, implemented in routine diagnostics.

The concept of Immune Risk Profile (IRP) is defined by the ratio of CD4 + T cells and CD8 + T cells (CD4/CD8 ratio) in combination with CMV seropositivity and, in some definitions, the expansion of terminally differentiated effector memory T cells [24, 25]. This profile is considered an indicator of immunosenescence and has demonstrated utility in predicting opportunistic infections following kidney transplantation [26, 27]. Other markers traditionally used to predict increased risk of opportunistic infections include low levels of T cell populations, typically measured one month after transplantation. Suggested thresholds are a total number of T cells less than 1000/µL, CD8 + T cells below100 /µL, or CD4 + T cells below50/µL [14, 15]. Similarly, a low number of NK cells (< 50/µL), measured 1 month after transplantation, has been associated with an increased risk of opportunistic infections in liver transplant recipients [16]. However, none of these studies have been able to identify patients at increased risk for opportunistic infections already at the time of transplantation.

The primary aim of this study was to assess the predictive value of two hypotheses regarding the risk of developing BKPyV replication: (a) whether the individual relative decline in CD4 + T cells 1–2 months after transplantation compared to pre-transplant levels is associated with increased risk, and (b) whether the concept of IRP can serve as a predictive marker. The secondary aim was to identify any immunological patterns that could help predict the risk of developing BKPyV replication after kidney transplantation, ideally already before transplantation.

## Methods

### Study design

This study included all consecutive adult (≥ 18 years) living kidney donors and their recipients who underwent transplantation at the Uppsala University Hospital, Sweden, between May 12, 2016, and September 24, 2018. The study was part of a single-centre prospective cohort study involving both deceased and living donor kidney transplant recipients [11]. The present study was restricted to recipients of living donor kidney transplants because additional blood samples for immune phenotyping were prospectively collected only in this subgroup. Analyses were based on recipients with available pre-transplant blood samples for immune cell phenotyping. All recipients were routinely screened for BKPyV DNA in plasma every 3 months for up to 18 months after transplantation, following the local screening program. For living donor kidney recipients, additional whole blood samples were collected for immune cell phenotyping at three time points: just before transplantation, 1–2 months after, and 6 months after transplantation. In addition, the living donors were sampled for blood immune cell phenotyping and provided urine samples for BKPyV DNA analysis at the time of transplantation, serving as a control group for the study. Although the study was prospectively designed with predefined sampling time points, follow-up samples were collected during routine clinical visits. Consequently, not all recipients contributed samples at every follow-up visit because of missed study visits, logistical reasons, or unavailable samples. Analyses at each time point were therefore performed using all available samples without imputation.

The study was approved by the Regional ethical review board in Uppsala (No. 2015/488). Clinical data were collected from electronic health records and the local transplantation database at the Uppsala University Hospital.

### BKPyV DNA analysis and BKPyVAN definition

BKPyV was analysed in plasma and urine samples using a modified variant of a previously described quantitative TaqMan real-time polymerase chain reaction (qPCR) assay, where the primers and probes were designed to give representative detection of all major genotypes [28]. Briefly, BKPyV DNA was extracted from 200 µL plasma or urine using the automatic NucliSens easyMAG robot (BioMérieux, Marcy l’Etoile, France). BKPyV DNA was then amplified from 5 µl out of 60 µl elution volume using TaqMan Universal PCR Master Mix (Thermo Fisher, Stockholm, Sweden) and the Qiagen Rotor-Gene Q thermo cycler (Qiagen, Hilden, Germany). The quantitative detection limit of the assay was determined to 400 copies/mL, and its proficiency was verified using external quality assessment programs from Instand (instandev.de) and Quality Control for Molecular Diagnostics (www.qcmd.org).

BKPyVAN was defined as positive immunohistochemical staining for Simian virus 40 large T antigen in a biopsy taken from the kidney allograft in combination with positive BKPyV DNAemia (≥400 copies/mL). At this center, biopsies were generally performed only for clinical indications, most commonly when patients experienced an unexplained increase in serum creatinine of at least 10%.

### Immune cell phenotyping

Samples analysed in this study were collected during clinical visits. An additional peripheral blood sample was obtained for research purposes to assess lymphocyte subsets. All flow cytometry analyses were performed on fresh whole blood.

Lymphocyte populations were quantified using the BD Multitest™ 6-Color TBNK reagent in combination with BD Trucount™ Tubes, a six-color direct immunofluorescence assay designed to identify and determine percentages and absolute counts of T cells, B cells, natural killer (NK) cells, and CD4 + and CD8 + T cell subpopulations. Briefly, 20 µL of Multitest antibody reagent were added to a Trucount tube, followed by 50 µL of whole blood. Samples were vortexed and incubated for 15 min in the dark. Subsequently, 450 µL of FACS™ Lysing Solution were added, followed by vortexing and an additional 15-minute incubation in the dark.

For detailed characterization of T- and B-cell subpopulations, two separate eight-color panels were used. The T-cell panel included antibodies to CD45, CD3, CD4, CD8, CD45RA, CCR7, HLA-DR, and CD38. The B-cell panel included antibodies to CD45, CD19, CD27, CD38, and CD24. Fluorochromes are listed in Supplementary Tables 1, and the corresponding immunophenotypic definitions of subpopulations in Supplementary Table 2. Samples were acquired on FACSCanto™ A and FACSCanto™ II cytometers (BD Biosciences). TBNK data were analysed with FACSDiva™ software (BD Biosciences), and T- and B-cell subsets with FlowJo™ v10 (BD Biosciences). Instrument setup, performance tracking with cytometer setup and tracking (CST) beads, and settings adjustments were performed according to the manufacturer’s recommendations.

Gating for the TBNK panel followed the manufacturer’s recommendations: lymphocytes were identified by side scatter (SSC) versus CD45, Trucount™ beads were identified by SSC versus their fluorescent channel, T cells were defined as CD3 + lymphocytes, CD8 + cytotoxic and CD4 + helper T cells were identified on CD8 vs. CD4 dot plots, B cells as CD3^−^CD16-CD56- lymphocytes expressing CD19, and NK cells as CD3^−^CD19^−^ lymphocytes expressing CD16 and/or CD56. Gating strategies for T- and B-cell subset panels are shown in Supplementary Fig. 1.

### Immune risk profile

Both donors and recipients were evaluated for IRP. Subjects who had a CD4/CD8 ratio < 1 and were seropositive for CMV, both before transplantation, were categorized as having a positive IRP.

### Classification of BKPyV replication

For the primary analyses, recipients were classified according to the presence or absence of BKPyV replication during follow-up, defined as detection of BKPyV DNA in plasma and/or urine. For descriptive analyses, participants with BKPyV replication were further classified according to the highest level of BKPyV replication detected during follow-up; (a) High-level BKPyV DNAemia, defined as plasma BKPyV DNA ≥10,000 copies/mL, consistent with presumptive BKPyV nephropathy according to the 2024 s International Consensus Guidelines [5, 29, 30]; (b) Low-level BKPyV DNAemia, defined as plasma BKPyV DNA ≥400-<10,000 copies/mL; and (c) BKPyV viruria only, defined as urine BKPyV DNA > 400 copies/mL in urine in the absence of detectable DNAemia.

### Immunosuppressive regimens

With few exceptions, patients received the local standard immunosuppressive regimen used during the study period, which consisted of induction therapy with anti-IL-2 receptor antibodies (basiliximab) and methylprednisolone, or methylprednisolone alone, as was considered standard of care (SOC) in Uppsala at the time of the study. Maintenance immunosuppression included tacrolimus administered twice daily and prednisolone in tapering doses. In addition, patients received mycophenolate mofetil (MMF).

Enhanced induction was defined as pretreatment with thymoglobulin and/or rituximab or eculizumab, often in combination with intravenous immunoglobulin (IVIg) and immunoadsorption and/or plasmapheresis/apheresis. Enhanced induction was given in certain situations, such as in the presence of HLA donor-specific antibodies, ABO incompatibility, and other higher risk immunological scenarios, including previous transplantations and high sensitization. The SOC induction regimen for ABO incompatibility was rituximab and glycosorb treatment, or in some cases, immunoadsorption and/or plasmapheresis. Rejection treatment included methylprednisolone, anti-thymocyte immunoglobulin, rituximab, eculizumab, IVIg and/or plasmapheresis.

In case of BKPyV DNAemia with low-level DNAemia, the locally applied recommendation was to increase the frequency of plasma DNA analysis to monthly intervals and to adjust immunosuppressive treatment. If high-level DNAemia was detected, follow-up was intensified and the MMF dose was reduced by 50%. If reducing the MMF dose was ineffective, MMF was discontinued and, in most cases, the tacrolimus dose was also reduced.

### Statistical analysis

Values were reported as means or medians with ranges. Differences between groups were tested using the non-parametric Mann–Whitney U test. Differences between more than two groups were tested by the means of the non-parametric Kruskal Wallis test and for analysing the specific sample pairs for stochastic dominance pairwise Mann–Whitney tests were used. For pairwise comparisons between results from the same recipient over time, the non-parametric Wilcoxon test was applied. Fisher’s exact test was used to test for statistical significance difference between group ratios.

P-Values below or equal to 0.05 were considered statistically significant. The statistical calculations were performed with GraphPad Prism version 10.2.3.

## Results

### Clinical Characteristics of Donors and Recipients

**Fig. 1:**
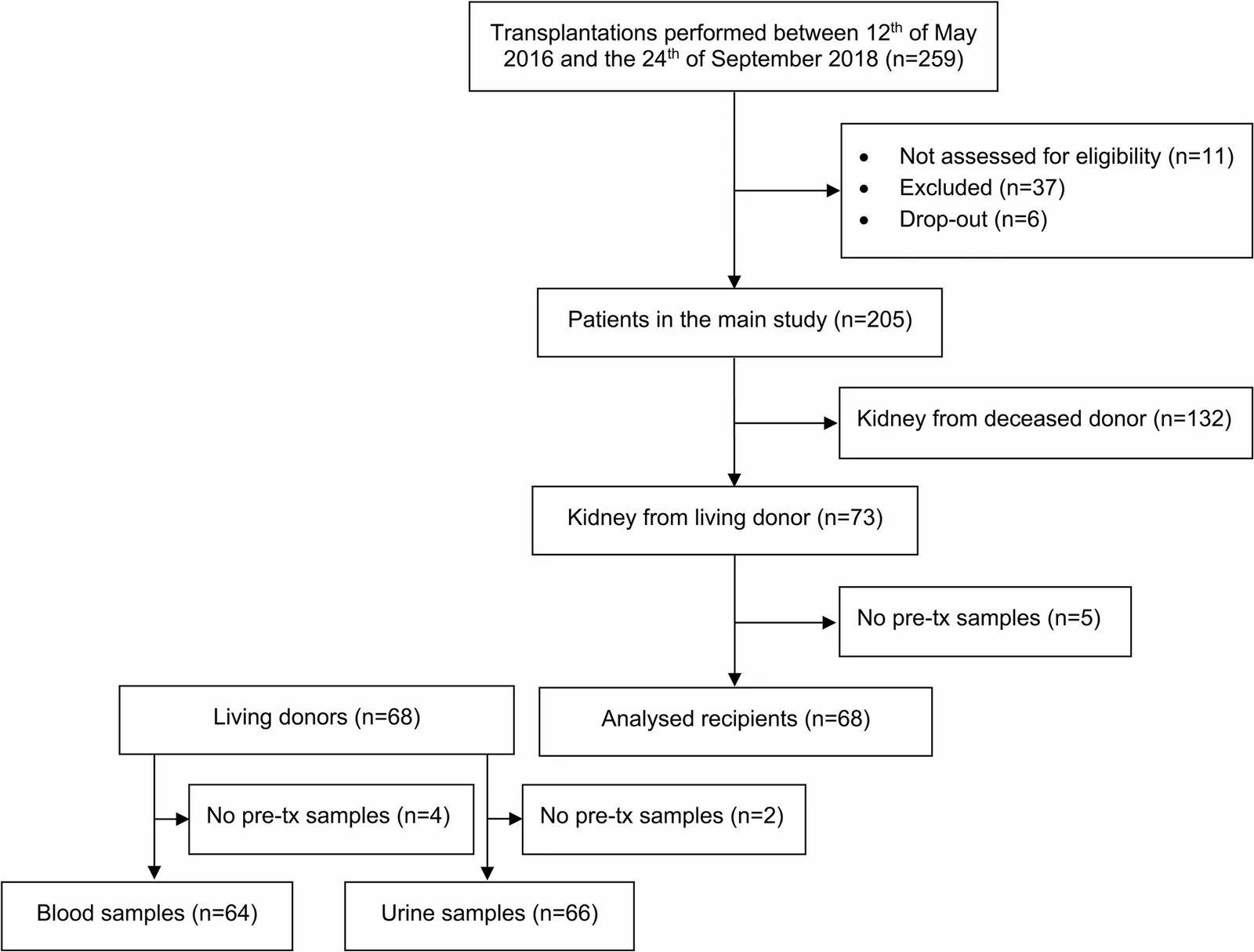
Flow chart of recipients and donors in the study. Pre-tx – Pre-transplant

During the study period, 259 patients were scheduled for kidney transplantation, and samples from 205 of these patients were included in the main study [11]. Among them, 73 recipients underwent living donor kidney transplantation. Of these, pre-transplant blood samples for immune cell phenotyping of CD4 + and CD8 + T cells, B cells and NK cells were available from 68 recipients (93.2%), who constituted the study population for the present analyses. Pre-transplant samples were not available from the remaining five recipients. A summary of the clinical setup is presented in Fig. 1. 

Samples were obtained from 44 recipients (64.7%) at 1–2 months after transplantation and from 45 recipients (66.2%) at 6 months after transplantation. Complete sampling at all three time points was achieved in 28 recipients (41.2%). In addition, samples for phenotyping of immune cell subtypes were collected from 40 recipients (59%) prior to transplantation, from 26 recipients (38%) at 1–2 months, and from 11 recipients (16%) at 6 months. Samples at all three time points were available for 2 recipients. From donors, 65 samples were collected for immune cell phenotyping of CD4 + and CD8 + T cells, B cells, and NK cells. In addition, samples for phenotyping of immune cell subtypes were collected from 38 donors. Missing follow-up samples were mainly due to unavailable study samples or missed follow-up visits rather than clinical outcomes, and analyses at each time point therefore included all recipients with available samples. Blood samples were collected from 64 donors and urine samples from 66 donors at the time of kidney donation.

A total of 21 recipients (30.9%) developed BKPyV replication in either plasma and/or urine. Of these, 11 (16,2%) developed BKPyV DNAemia, including eight recipients (11.8%) with high-level DNAemia and three (4.4%) with low-level DNAemia, while 10 (14.7%) had BKPyV viruria only. One recipient (1.5%) developed BKPyVAN. Mean time to first BKPyV DNA detection was 3.9 months (range 1.5–6 months). Eight donors (12.1%) had BKPyV viruria. Patient and donor characteristics are presented in Table 1.

**Table 1:** Demographics and clinical characteristics of recipients and donors. Kidney recipients with and without BKPyV replication. The table also shows a subgroup analysis of BKPyV DNA-positive recipients according to BKPyV DNA level. Categorical variables are presented as n (%), and quantitative variables are presented as median (range). The number of patients with rejection treatment in this table is both prior to, concurrent with, and after developing BKPyV replication

Characteristic	Total (n=68)	No BKPyV (n=47)	BKPyV (n=21)	High DNAemia (n=8)	Low DNAemia (n=3)	Viruria only (n=10)
Blood samples						
Samples at 1–2 months	44 (64.7)	27 (57.4)	17 (81.0)	7 (87.5)	2 (66.7)	7 (70.0)
Samples at 6 months	45 (66.2)	32 (68.1)	13 (61.9)	5 (62.5)	1 (33.3)	7 (70.0)
Samples at all 3 time points	28 (41.2)	17 (36.2)	11 (52.4)	5 (62.5)	1 (33.3)	5 (50.0)
BKPyVAN	1 (1.5)		1 (4.8)	1 (12.5)	0	0
Male sex	41 (60.3)	26 (55.3)	15 (71.4)	6 (75.0)	1 (33.3)	8 (80.0)
Age (years)	52 (20–73)	53 (20–73)	52 (23–68)	56 (30–68)	23 (23–42)	52 (25–68)
Weight (kg)	77 (50–124)	78 (50–124)	73 (56–98)	84 (56–98)	65 (64–81)	62 (57–97)
Height (cm)	174 (155–198)	173 (155–198)	175 (157–191)	176 (159–179)	175 (167–187)	175 (157–191)
BMI	25.2 (17.3–34.3)	25.1 (17.3–34.3)	25.3 (18.6–32.4)	27.8 (21.1–31.3)	20.9 (19.6–29.0)	24.0 (20.1–32.4)
Diabetes	10 (14.7)	7 (14.9)	3 (14.3)	1 (12.5)	1 (33.3)	1 (10.0)
Primary cause of renal failure						
Glomerulonephritis	20 (29.4)	15 (31.9)	5 (23.8)	1 (12.5)	1 (33.3)	3 (30.0)
Miscellaneous/unknown	18 (26.5)	11 (23.4)	7 (33.3)	3 (37.5)	0	4 (40.0)
Cystic/Hereditary/Congenital	11 (16.2)	8 (17.0)	3 (14.3)	1 (12.5)	0	2 (20.0)
Diabetes	8 (11.8)	6 (12.8)	2 (9.5)	1 (12.5)	1 (33.3)	0
Hypertension/Large vessel disease	9 (11.8)	6 (12.8)	2 (9.5)	1 (12.5)	0	1 (10.0)
Interstitial nephritis/pyelonephritis	3 (4.4)	1 (2.1)	2 (9.5)	1 (12.5)	1 (33.3)	0
Number of transplants						
First tx	59 (86.8)	43 (91.5)	16 (76.2)	6 (75.0)	2 (66.7)	8 (80.0)
Second tx	8 (11.8)	4 (8.5)	4 (19.0)	2 (25.0)	0	4 (40.0)
Third or more tx	1 (1.5)	0	1 (4.8)	0	1 (33.3)	0
Donor age (years)	50 (24–72)	49 (24–72)	52 (26–66)	55 (45–66)	52 (50–55)	52 (26–65)
Donor male sex	28 (41.2)	20 (42.6)	8 (38.1)	1 (12.5)	1 (33.3)	6 (60.0)
Recipient CMV+	48 (70.6)	33 (70.2)	15 (71.4)	7 (87.5)	1 (33.3)	7 (70.0)
Donor CMV+	49 (72.1)	33 (70.2)	16 (76.2)	8 (100.0)	2 (66.7)	6 (60.0)
CMV mismatch (D+/R-)	10 (14.7)	7 (14.9)	3 (14.3)	1 (12.5)	1 (33.3)	1 (10.0)
Enhanced induction	23 (33.8)	18 (38.3)	5 (23.8)	3 (37.5)	1 (33.3)	1 (10.0)
Rituximab or Eculizumab	19 (27.9)	14 (29.8)	3 (14.3)	1 (12.5)	1 (33.3)	1 (10.0)
Thymoglobulin	0	0	0	0	0	0
IVIg	2 (2.9)	0	2 (9.5)	0	1 (33.3)	1 (10.0)
Immunoadsorption/plasmapheresis	6 (8.8)	4 (8.5)	2 (9.5)	1 (12.5)	1 (33.3)	0
Glycosorb	18 (26.5)	15 (31.9)	3 (14.3)	2 (25.0)	0	1 (10.0)
ABO incompatible	19 (27.9)	15 (31.9)	4 (19.0)	3 (37.5)	0	1 (10.0)
Day of onset of graft function (d)	1.4	1.3	1.4	2.0	1.0	1.0
Rejection treatment	12 (17.6)	9 (19.1)	3 (14.3)	1 (12.5)	0	2 (20.0)
Solumedrol	12 (17.6)	9 (19.1)	3 (14.3)	1 (12.5)	0	2 (20.0)
Thymoglobulin	4 (5.9)	4 (8.5)	0	0	0	0
Eculizumab	1 (1.5)	1 (2.1)	0	0	0	0
Graft loss or death	5 (7.4)	3 (6.4)	2 (9.5)	1 (12.5)	0	1 (10.0)
Death	2 (2.9)	1 (2.1)	1 (4.8)	0	0	1 (10.0)

### Immune risk profile as a predictor of BKPyV replication

When assessing the immune risk profile, 17 of the recipients (25.0%) and one donor (1.5%) met the criteria for IRP-positivity. All recipients and donors with a CD4/CD8 ratio < 1 were serologically positive for CMV. Among the IRP-positive recipients, nine developed BKPyV replication (52.9%), indicating a higher risk compared to the IRP-negative recipients (OR 3.66, CI 1.08–10.79, *p* = 0.034). Of these nine IRP-positive recipients, five were positive in plasma, four of whom had high-level BKPyV DNAemia, and four had BKPyV viruria only.

The donor with IRP-positivity also had detectable BKPyV DNA in urine, and the corresponding recipient later developed high-level BKPyV DNAemia.

### Immune cell counts before transplantation

**Fig. 2:**
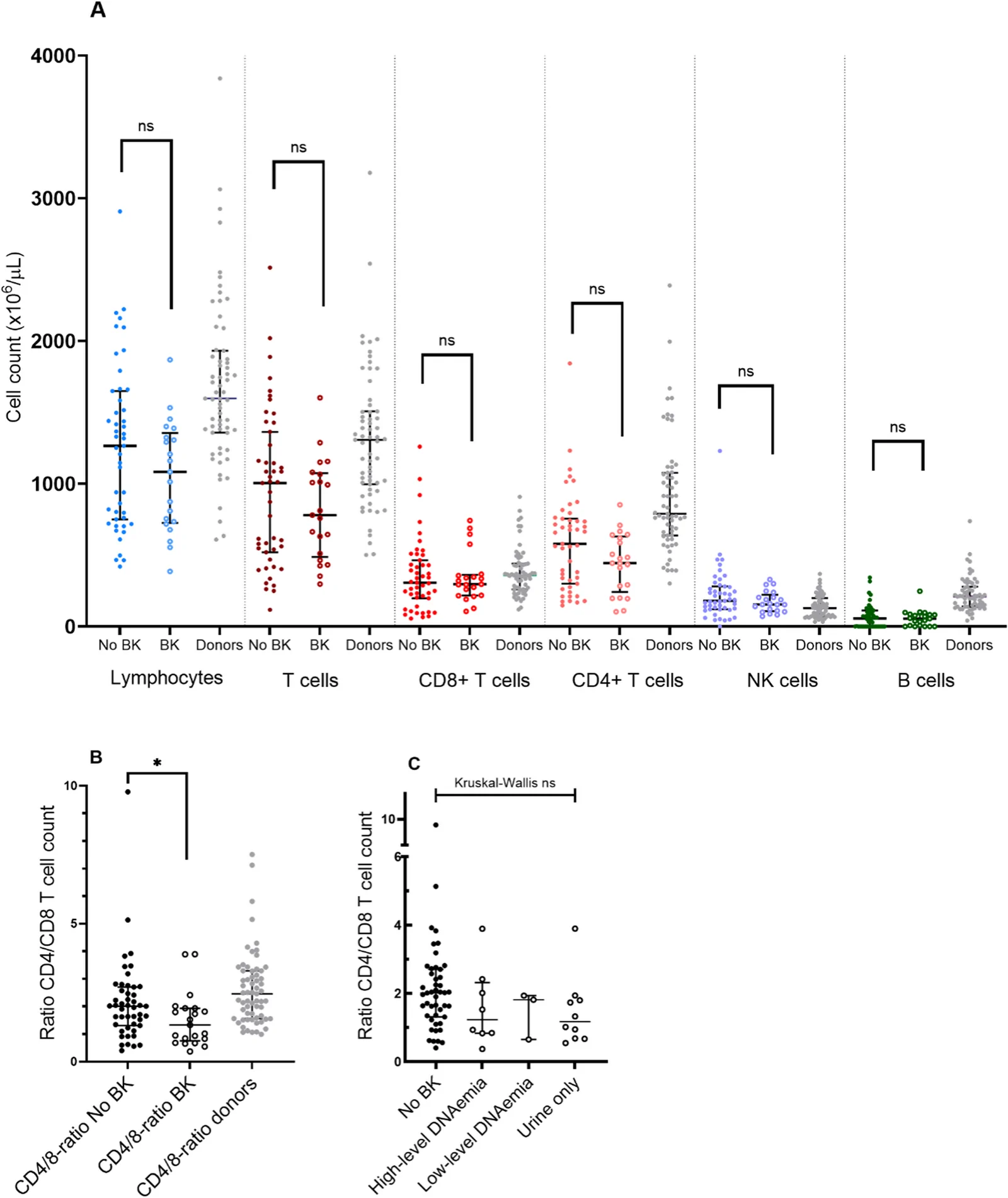
**A** Lymphocyte count in blood, before transplantation, from recipients with and without BKPyV replication detectable in plasma or urine, and in donors. **B** CD4/CD8 ratio in blood, before transplantation, from recipients with and without BKPyV replication detectable in plasma or urine, and in donors. **C** CD4/CD8 ratio in blood, before transplantation, from recipients with and without BKPyV replication detectable in plasma or urine, divided by BKPyV DNA level. Dots represent individual values; median and interquartile range are indicated by lines

There was no difference in lymphocyte counts between recipients who later developed BKPyV replication and those who did not (Fig. 2A). However, the CD4/CD8 ratio before transplantation was lower in recipients who subsequently developed BKPyV replication compared to those who did not (*p* = 0.03, Fig. 2B). Among recipients who developed BKPyV replication, further analyses by virus level showed no differences between the groups (Fig. 2C).

There were no differences in T or B cell subpopulation counts before transplantation between patients who later developed BKPyV replication and those who did not (Supplementary Fig. 2). The number of activated T cells was too low for reliable assessment. Recipients had lower total lymphocyte counts (*p* < 0.0001), T cells (*p* < 0.0001), CD4 + T cells (*p* < 0.0001), B cells (*p* < 0.0001) and lower CD4/CD8 ratios (*p* = 0.0001) before transplantation compared to donors (Supplementary Fig. 3). In contrast, recipients had higher NK cell counts (*p* = 0.003) than donors prior to transplantation. The difference in cell counts between recipients and donors were even more pronounced among those who had received enhanced induction therapy. No differences were observed in any cell population between donors to recipients with BKPyV replication in plasma or urine and donors to recipients without detectable BKPyV replication.

### Immune cell counts after transplantation

**Fig. 3:**
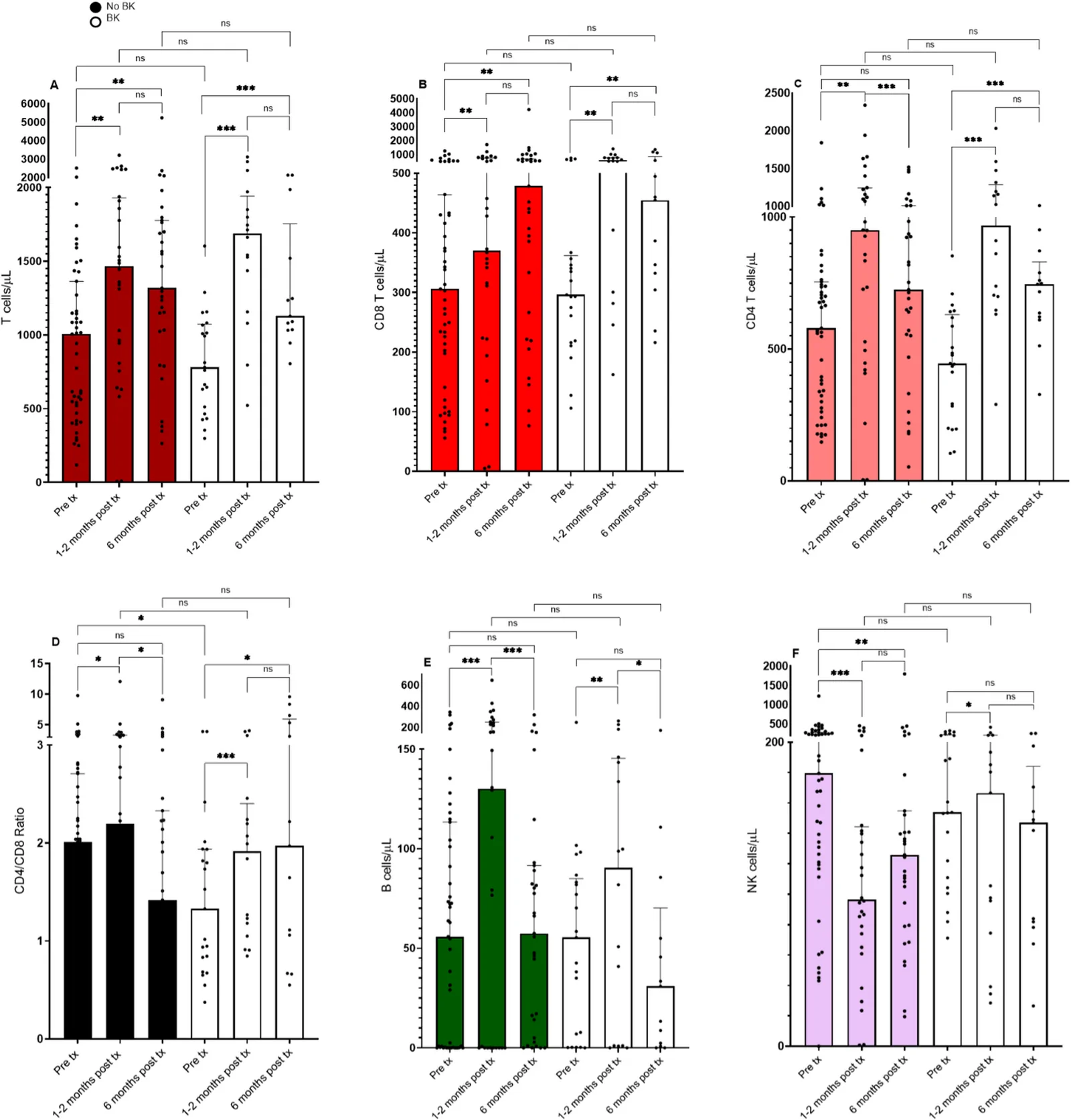
Cell counts pre-transplant (pre-tx), 1–2 months and 6 months post-transplant (post-tx) for **A** T cells, **B** CD8 + T cells, **C** CD4 + T cells, **D** CD4/CD8 ratio, **E** B cells and **F** NK cells, grouped by no BKPyV replication (filled bars) and BKPyV replication (open bars). Dots represent individual values. Bars show medians. Error bars indicate the interquartile range

The total number of T cells, as well as CD8 + and CD4 + T cell counts, increased 1–2 months post-transplantation compared to pre-transplantation levels in both recipients who developed BKPyV replication (T cells *p* = 0.0002, CD8 + T cells *p* = 0.001, CD4 + T cells *p* = 0.0002) and those who did not (T cells *p* = 0.002, CD8 + T cells *p* = 0.004, CD4 + T cells *p* = 0.004) (Fig. 3A-C). However, although recipients who developed BKPyV replication showed a greater increase in CD4 + T cell counts at both 1–2 months and 6 months post-transplantation, this difference was not statistically significant. (Supplementary Fig. 4). While total T cell and CD8 + T cell count remained elevated 6 months post-transplantation in both groups, the CD4 + T cell count returned to baseline levels by 6 months in patients who did not develop BKPyV replication. The CD4/CD8 ratio increased slightly 1–2 months post-transplant in both groups (*p* = 0.0006 in recipients who developed BKPyV replication and *p* = 0.02 in those who did not). At 6 months post-transplantation, the ratio decreased again in recipients who did not develop BKPyV replication (*p* = 0.02)., while no statistically significant difference in CD4/CD8 ratio was observed in recipients who did develop BKPyV replication (Fig. 3D). The B cell count increased 1–2 months after transplantation but returned to pre-transplant levels by the 6-month time point in both recipients who developed BKPyV replication (*p* = 0.001 at 1–2 months, *p* = 0.03 at 6 months) and those who did not (*p* = 0.0009 at 1–2 months, *p* = 0.0006 at 6 months) (Fig. 3E). Both naïve and memory B cells contributed to this transient increase in B-cells (Supplementary Fig. 2). The number of NK cells decreased after 1–2 months post-transplant (*p* = 0.0007) and remained at similar levels at 6 months (*p* = 0.002) in recipients who did not develop BKPyV replication. In recipients who developed BKPyV replication, there was an increase in NK cell count at 1–2 m (*p* = 0.02, Fig. 3F). T and B cell subpopulation analysis showed similar distributions in both patients who developed BKPyV replication and those who did not (Supplementary Fig. 2). The time span for the first follow-up sampling was 1 to 2 months after transplantation. When limiting the analysis to samples collected 1 month post-transplant, the pattern was similar for all cell lines.

Results from immune cell phenotyping in recipients, grouped according to BKPyV DNA levels in plasma and urine, are presented in Supplementary Fig. 5.

Previously proposed thresholds for total T cell, CD8 + T cell, CD4 + T cell, and NK-cell counts at transplantation and 1–2 months post-transplant did not show any statistically significant association with subsequent BKPyV replication in our cohort (all p-values > 0.05). No clear correlation was observed for low T cell or NK cell count at either time point.

## Discussion

In this prospective study, we analysed the relationship between BKPyV replication and the cellular immune response in recipients of kidneys from living donors. Previous studies have shown that a decrease in BKPyV-specific CD4 + T cells 1–2 months post-transplantation is associated with an increased risk of BKPyV infection after kidney transplantation [9, 17, 18]. Our primary aim was to determine whether this association also applies to total CD4 + T cell counts, which are analysed in routine diagnostics, and to assess whether immune risk profiling could help predict the risk of subsequent BKPyV replication. The secondary aim was to improve the efficiency of BKPyV screening by evaluating the potential of immune cell populations as indicators for identifying recipients at higher risk of BKPyV replication.

When comparing the recipients who subsequently developed BKPyV replication to those who did not, there were no differences in cell counts before transplantation, but the CD4/CD8 ratio was lower in recipients who later developed BKPyV replication. This suggests a potential association between a lower CD4/CD8 ratio and increased risk of clinically significant BKPyV replication and supports the relevance of the immune risk profile (IRP) concept in this setting. However, there was a substantial overlap between the groups, limiting the utility of this concept for individualized screening purposes. Although lymphocyte immunophenotyping by flow cytometry is available in many transplant centres internationally, its role as a routine tool for risk stratification after kidney transplantation remains uncertain. In our study, the observed overlap in immune cell profiles between recipients with and without BKPyV replication limits the feasibility of implementing lymphocyte typing as a routine approach for individual BKPyV risk prediction. This overlap persisted whether recipients were grouped simply by presence or absence of BKPyV replication or further subdivided into clinically more relevant categories [29, 30], i.e. high-level BKPyV DNAemia, low-level DNAemia, isolated BKPyV replication in the urine, or BKPyV DNA negative. For most analyses, we used the binary classification of BKPyV replication versus non-replication to ensure sufficient sample sizes. This approach is also consistent with the 2024 s International Consensus Guidelines, which recognize persistent plasma BKPyV DNAemia between 1,000 and 10,000 copies/mL as clinically relevant and recommend reduction of immunosuppression for persistent low-level DNAemia as well as for DNAemia exceeding 10,000 copies/mL [5, 31], thereby supporting the clinical relevance of including both low- and high-level BKPyV DNAemia in our analyses. Notably, all recipients with a CD4/CD8 ratio < 1 were CMV seropositive, thereby meeting the CMV serostatus criterion of the IRP. In this cohort, CMV serostatus therefore did not add additional discriminatory value beyond the CD4/CD8 ratio within this subgroup, suggesting that the CD4/CD8 ratio may largely explain the observed association with BKPyV replication in this setting.

Although no statistically significant differences in baseline lymphocyte, total T-cell, or CD4 + T-cell counts were observed between recipients who subsequently developed BKPyV replication and those who did not, Fig. 2A suggests numerically higher baseline values for these cell populations in recipients who remained free of BKPyV replication. Given the relatively small cohort size, particularly the limited number of recipients who developed BKPyV DNAemia, the study may have had insufficient statistical power to detect modest differences between groups. Consequently, we cannot exclude the possibility that baseline differences in immune cell counts contribute to the risk of subsequent BKPyV replication, and this question warrants further investigation in larger cohorts.

Our findings did not support the hypothesis that a relative decline in total CD4 + T cells from pre-transplant to 1–2 months post-transplant increases the risk of BKPyV replication. Conversely, all lymphocyte populations increased in most recipients during the first months after transplantation, including patients who developed BKPyV replication. The lack of association between total CD4 + T cell count and risk of BKPyV replication is likely because BKPyV-specific T cells constitute only a relatively small fraction of the total T cell population. In addition, BKPyV serostatus was not included in the present analyses. Consequently, we could not assess whether pre-existing BKPyV immunity influenced the relationship between total T-cell counts and BKPyV-specific cellular immunity. Schachtner et al. however, described a decrease not only in BKPyV specific T cells but also in total lymphocytes (CD3 + cells) and total CD4 + T cell counts during the first months after transplantation in recipients who developed BKPyV replication, particularly among ABO-incompatible kidney transplant recipients [14, 20]. Fernández-Ruiz et al. also identified low CD8 + and CD4 + T cells as risk factors for opportunistic infections in kidney recipients [15]. In another study, the same group presented low NK cells as a risk factor for opportunistic infections in liver transplant recipients [16]. However, none of these studies could identify patients at increased risk of opportunistic infections already at the time of transplantation. In our study, we were unable to confirm any of the previously proposed thresholds for total T cell, CD8 + or CD4 + T cell, or NK cell counts, neither before transplant nor 1–2 months after. We cannot fully explain these contradictory results, as our cohort was comparable in size and immunosuppression protocols to previous studies. Although we allowed a wider time interval (1–2 months) for the first follow-up sampling compared to the stricter 1 month window in other studies, restricting our analysis to only include samples collected 1 month after transplantation, yielded similar results.

In total, 31% of the recipients developed BKPyV replication; 16% had BKPyV DNAemia, with 12% experiencing high level DNAemia, and one recipient (1.5%) was diagnosed with BKPyVAN. These findings are consistent with previously reported incidence rates in kidney recipients from living donors, where the incidence of BKPyV DNAemia has varied between 12 and 29%, and the incidence of BKPyVAN between 0.8 and 10% [32–34]. While BKPyV DNAemia rates are comparable across kidney transplant populations, including living donor cohorts, the incidence of BKPyV-associated nephropathy in our study remained low. This suggests that factors other than donor type, such as host immune response, may play a more important role in progression to clinically significant disease.

We found that, before transplantation, recipients generally had lower cell counts of total lymphocytes, CD4 + T cells, and B cells compared to donors. In contrast, CD8 + T cell levels were similar between the groups, while NK cell counts were higher in recipients than in donors. These differences in immunological status may be partly explained by pre-transplant immunosuppressive therapy in some recipients prior to sampling, e.g. those receiving an ABO incompatible organ. Additionally, underlying diseases and previous transplants may have contributed to these variations.

CD4 + and CD8 + T cell counts, as well as B cell counts, increased 1–2 months after transplantation. This likely reflects early immune reconstitution following transplantation and redistribution of lymphocyte subsets after engraftment, in the context of ongoing immunosuppressive therapy.

We also explored whether donor immune cell parameters were associated with subsequent BKPyV replication in the corresponding recipients. No differences were observed in lymphocyte counts, T and B cell populations, NK cell counts, or CD4/CD8 ratios between donors to recipients with and without BKPyV replication. However, given the limited sample size, we cannot exclude modest donor-related effects, and larger studies are needed to further evaluate the potential contribution of donor immune status to recipient susceptibility to BKPyV replication.

A strength of this study is its prospective design, with blood samples collected at predetermined time points and a cohort size comparable to other studies, both in terms of patient numbers and incidence of DNAemia. Another strength is the inclusion of controls from the recipients themselves, with pre- and post-transplantation comparisons, as well as healthy donor controls. The study also benefited from the relatively homogeneous living donor transplant population, reducing variability related to donor characteristics and perioperative factors. Limitations include the fact that follow-up samples were only obtained from approximately 65% of recipients. Although sampling was prospectively planned, follow-up samples were collected during routine clinical visits, resulting in some missing samples at later time points. Analyses were therefore performed using all available samples at each time point. The relatively small cohort size, and particularly the limited number of recipients who developed BKPyV replication, may have limited the statistical power to detect modest differences between groups. For example, although not reaching statistical significance, the observed trend toward higher baseline lymphocyte counts, total T cell counts, and CD4 + T cell counts in recipients who did not subsequently develop BKPyV may reflect biological variation that could not be fully captured in this cohort. Larger studies are needed to clarify whether such baseline immunological differences contribute to susceptibility to BKPyV replication after kidney transplantation. Another limitation is that only recipients of living donor kidney transplants were included. While this provided a clinically homogeneous study population, it may limit the generalisability of our findings to recipients of deceased donor kidneys, who represent the majority of kidney transplant recipients. Future studies including both living and deceased donor recipients would be valuable to increase statistical power and improve the generalisability of these findings. Additionally, precise data on immunosuppressive dosing were not available, limiting comparisons of immunosuppression with other studies. Finally, analysing BKPyV-specific T cells alongside total lymphocyte numbers could have provided deeper insights into the relationship between immune status and the risk of BKPyV replication.

## Conclusion

In conclusion, this prospective study suggests an association between pre-transplant IRP-positivity, defined by a low CD4/CD8 ratio in combination with CMV seropositivity, and subsequent BKPyV replication after transplantation. However, substantial overlap between groups limits the clinical utility of these markers for individual risk prediction.

No association was observed between absolute lymphocyte counts or their subpopulations and subsequent BKPyV replication, and, contrary to our hypothesis, most lymphocyte subsets increased after transplantation regardless of infection outcome.

Overall, IRP showed a possible association with BKPyV replication, whereas other routinely available lymphocyte-based parameters lacked sufficient discriminatory value. Further studies are needed to determine whether these or other immune biomarkers may improve early identification of kidney transplant recipients at risk of developing BKPyV replication.

## Supplementary Information

Supplementary Material 1.

## Data Availability

The datasets used and/or analysed during the current study are available from the corresponding author on reasonable request.
