# Supplementary material for: Cellular immune response during BK polyomavirus infection in living donor kidney transplant recipients
Source: BMC Immunol. 2026 Sep 26;27:92. doi: 10.1186/s12865-026-00912-x (PMC13660182; doi:10.1186/s12865-026-00912-x)
Supplement: Supplementary file 1 [file 12865_2026_912_MOESM1_ESM.docx]

**SUPPLEMENTARY MATERIAL**

**Supplementary Table 1.** Fluorochromes used in flow cytometry panels.

| **Fluochrome** | **Marker** | **Cell type** | **Antibody clone** | **Isotype** | **Supplier** |
| --- | --- | --- | --- | --- | --- |
| FITC | CD45+ | Leukocytes | HI30 | Mouse IgG1, κ | BD Biosciences |
| V450-A | CD3+ | T-lymphocytes | UCHT1 | Mouse IgG1, κ | BD Biosciences |
| PerCP-Cy5.5-A | CD4+ | T-cell subset | Leu-3a | Mouse IgG1, κ | BD Biosciences |
| APC-H7-A | CD8+ | T-cell subset | SK1 | Mouse IgG1, | BD Biosciences |
| APC-A | CD38+ | Activation marker | HB7 | Mouse IgG1, κ | BD Biosciences |
| V500-A | HLA-DR | Activation marker | G46-6 | Mouse IgG2a, κ | BD Biosciences |
| PE-A | CCR7+/CD197 | Naive T-cells | 150503 | Mouse IgG2a | BD Biosciences |
| PE-Cy7-A | CD45RA | Naive T-cells/ TEMRA | HI100 | Mouse IgG2b, κ | BD Biosciences |
|  |  |  |  |  |  |
| FITC | CD45+ | Leukocytes | HI30 | Mouse IgG1, κ | BD Biosciences |
| V450-A | CD3+ | T-lymphocytes | UCHT1 | Mouse IgG1, κ | BD Biosciences |
| PerCP-Cy5.5-A | CD19+ | Pan-B cell marker | HIB19 | Mouse IgG1, κ | BD Biosciences |
| APC-H7-A | CD20+ | Pan-B cell marker | 2H7 | Mouse IgG2b, κ | BD Biosciences |
| PE-Cy7-A | CD27+ | Memory B-cells | M-T271 | Mouse IgG1, κ. | BD Biosciences |
| V500-A | IgD+ | Immature/ Naive marker | IA6-2 | Mouse IgG2a, κ. | BD Biosciences |
| PE-A | CD24+ | Translational B cells | ML5 | Mouse IgG2a, κ | BD Biosciences |
| APC-A | CD38+ | Plasmablasts | HB7 | Mouse IgG1, κ | BD Biosciences |

**Supplementary table 2.** Immunophenotypic definitions of T- and B-cell subpopulations.

| **Cell population** | **Definition** |
| --- | --- |
| Naive CD4+ T-cells | CD3+CD45RA+CCR7+ |
| Central memory T cells | CD3+CD45RA-CCR7+ |
| Effector memory T cells | CD3+CD45RA-CCR7- |
| Effector memory RA+ T cells (T_EMRA_) | CD3+CD45RA+CCR7- |
|  |  |
| Naive B-cells | CD19+CD20+CD27-IgD+ |
| Memory B-cells | CD19+CD27+IgD+ |
| Translational B-cells | CD19+CD24++CD38++ |
| Plasmablast | CD19+CD27++CD38++ |

**Supplementary Figure 1.** Gating strategies for T- and B-cell subsets.

**Supplementary Figure 2.** Cell counts of T and B cell subpopulations before kidney transplantation, 1-2 months post-transplant and 6 months post-transplant, in recipients who later developed BKPyV (open bars) or not (black bars) and in donors (grey bars). Dots represent individual values. Bars show medians. Error bars indicate the interquartile range. EMRA, terminally differentiated effector memory cells re-expressing CD45RA; Ns, not significant.

**Supplementary Figure 3.** Immune cell counts (A) and CD4/CD8 T-cell ratio (B) in kidney donors and recipients with and without enhanced induction therapy. Dots represent individual values. Bars show medians. Error bars indicate the interquartile range.

**Supplementary Figure 4.** Difference in CD4+ T cell numbers between pre-transplantation samples and 1-2 months (A) and 6 months (B), respectively. Patients with high-level DNAemia are depicted in orange, patients with low-level viremia in beige, and patients with BKPyV DNA detectable only in urine are depicted in green. Dots represent individual values. Bars show medians. Error bars indicate the interquartile range.

**Supplementary Figure 5.** Immune cells in recipients who developed high titer BKPyV DNAemia (orange), low-titer DNA BKPyV DNAemia (beige), BKPyV DNA detectable only in urine (green), and in recipients with no detectable BKPyV DNA levels (open bars). Dots represent individual values. Bars show medians. Error bars indicate the interquartile range.
